# Unique nonstructural dysfunction of the omnicarbon valve: case report

**DOI:** 10.1186/s13019-016-0436-9

**Published:** 2016-03-29

**Authors:** Soki Kurumisawa, Yuichiro Kaminishi, Arata Muraoka, Yoshio Misawa

**Affiliations:** Division of Cardiovascular Surgery, Jichi Medical University, 3311-1 Yakushiji, Shimotuske, Tochigi 329-0498 Japan

**Keywords:** Mitral valve replacement, Omnicarbon valve, Pannus formation

## Abstract

**Background:**

Pannus formation may disturb the leaflet movement of the prosthetic valve.

**Case presentation:**

A 61-year-old woman presented with exertional dyspnea. She had undergone mitral valve replacement with a bioprosthetic valve 31 years ago, which was replaced with a tilting disc valve 22 years ago. The present laboratory findings revealed hemolytic anemia. Echocardiography showed an increased mean pressure gradient through the mitral valve and moderate to severe regurgitation around the minor orifice of the tilting disc valve. She therefore underwent a third operation. Pannus formation was found on the prosthetic valve ring, but it did not obliterate the prosthetic valve orifice. After removing the valve, the posterior wall of the left ventricle was seen to be associated with thickened endocardium. A bileaflet valve was implanted. Postoperative echocardiography showed that the implanted valve functioned well.

**Conclusions:**

Nonstructural dysfunction of the mechanical heart valve might occur long after operation. These changes are particularly observed with a tilting disc valve.

**Electronic supplementary material:**

The online version of this article (doi:10.1186/s13019-016-0436-9) contains supplementary material, which is available to authorized users.

## Background

Nonstructural dysfunction of the mechanical heart valve may be caused by paravalvular leak, prosthetic valve endocarditis, and/or pannus formation. The latter may disturb the leaflet movement of the prosthesis and may grow beyond the suture ring. We describe a patient who was diagnosed with mitral valve stenosis and regurgitation 22 years after her implanted bioprosthetic valve was replaced with an Omnicarbon valve (Medical Incorporated, Minneapolis, MN, USA).

## Case presentation

A 61-year-old woman presented with a 10-month history of exertional dyspnea. She had had rheumatic heart valve disease and had undergone mitral valve replacement with a Carpentier Edwards valve (Edwards Lifesciences, Irvine, CA, USA) without preservation of the posterior leaflet of the mitral valve 31 years ago. Because of primary tissue failure of this implanted valve, she underwent valve replacement for with an Omnicarbon valve 22 years ago. During this second replacement, the bioprosthetic valve was removed, and the Omnicarbon valve was implanted in an intra-annular position, with the major orifice orientated posteriorly. Our standard method then was a U-suture technique using 2-0 sutures with pledgets. Controlled Anticoagulation therapy combined with paramidine was applied postoperatively.

At the present admission, laboratory findings revealed a white blood cell count of 2100/μl, hemoglobin of 9.7 g/dl, and slight elevations in the serum levels of aspartate aminotransferase (54 mU/ml), lactate dehydrogenase (1277 mU/ml), and total bilirubin (2.65 mg/dl). Brain natriuretic peptide was also elevated (100.8 pg/ml).

**Fig. 1:**
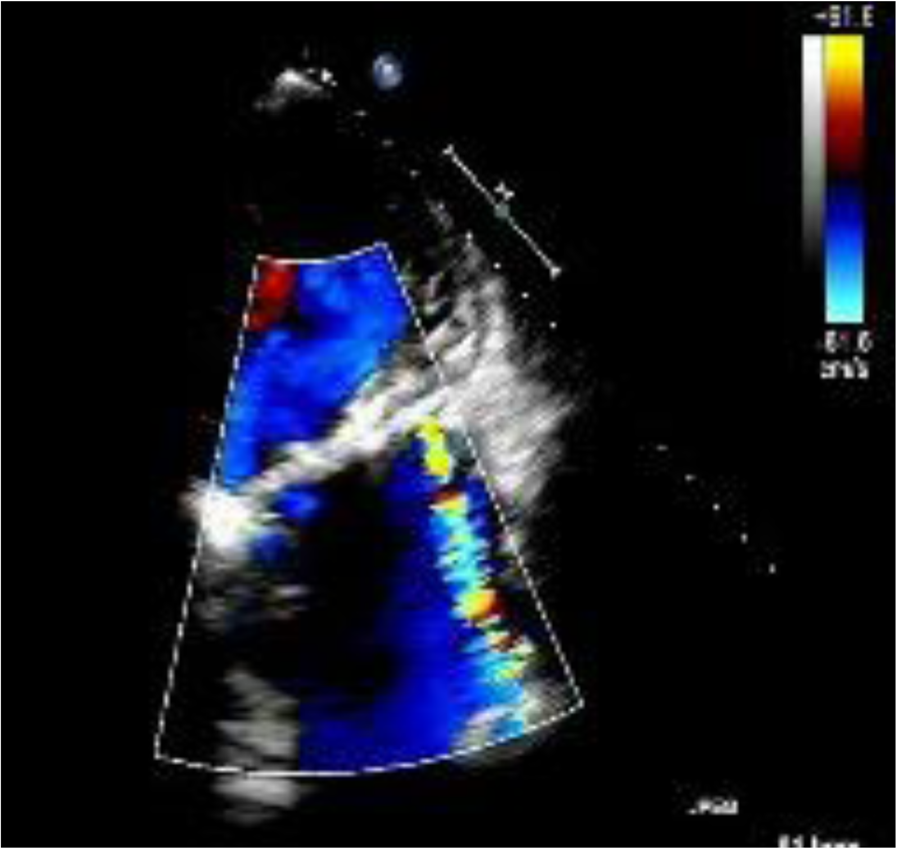
Preoperative echocardiographic findings of a long axis view. Mitral valve regurgitation is apparent around the minor orifice of the implanted valve. The left atrium is enlarged

**Fig. 2:**
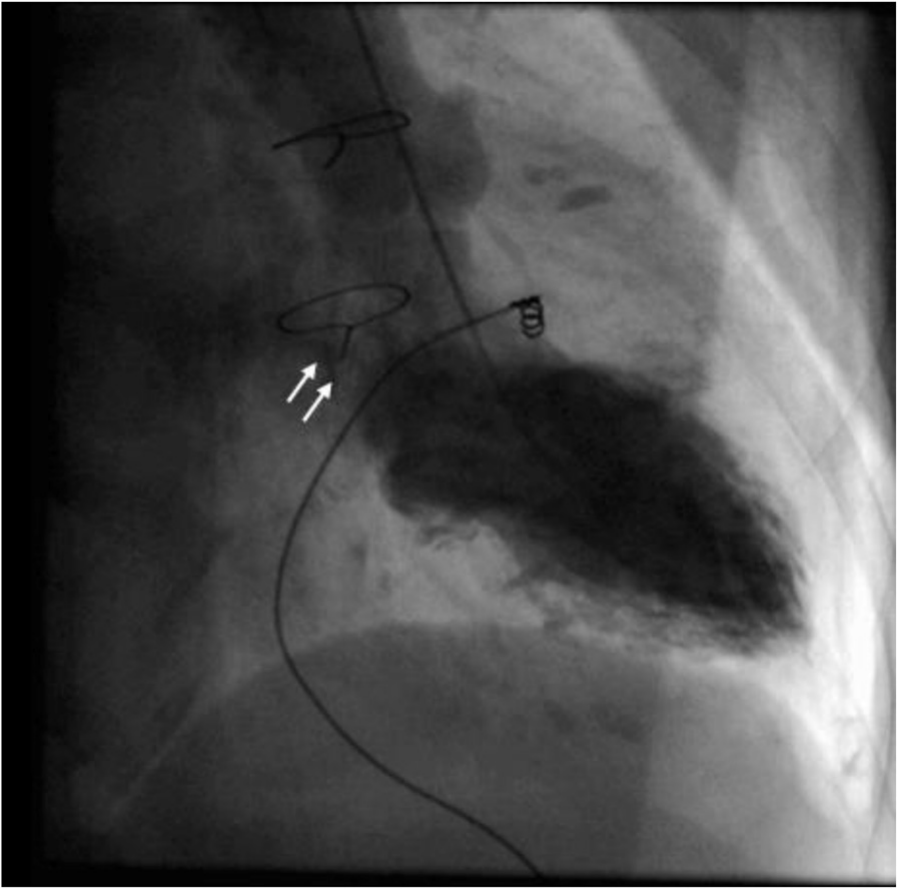
Left ventriculographic findings. Mitral valve regurgitation from the minor orifice of the Omnicarbon valve is observed (*white arrows*)

Chest radiography showed a high cardiothoracic ratio of 86 % with right pleural effusion. Electrocardiography revealed atrial fibrillation with an extremely low f wave. Trans-thoracic echocardiography showed that the peak flow velocity of the Omnicarbon valve was 2.7 m/s, the mean pressure gradient between the left atrium and ventricle was 15 mmHg, and moderate-to-severe mitral valve regurgitation around from the minor orifice of the Omnicarbon valve (Fig. 1) with an enlarged left atrium (80 mm). The left ventricular ejection fraction was 60 % with left ventricular end-diastolic/end-systolic dimensions of 47/34 mm. Preoperative computed tomography also showed the enlarged left atrium and right pleural effusion. Left ventriculography disclosed that mitral valve regurgitation from the minor orifice of the Omnicarbon valve and the enlarged left atrium (Fig. 2, Additional file 1).

**Fig. 3:**
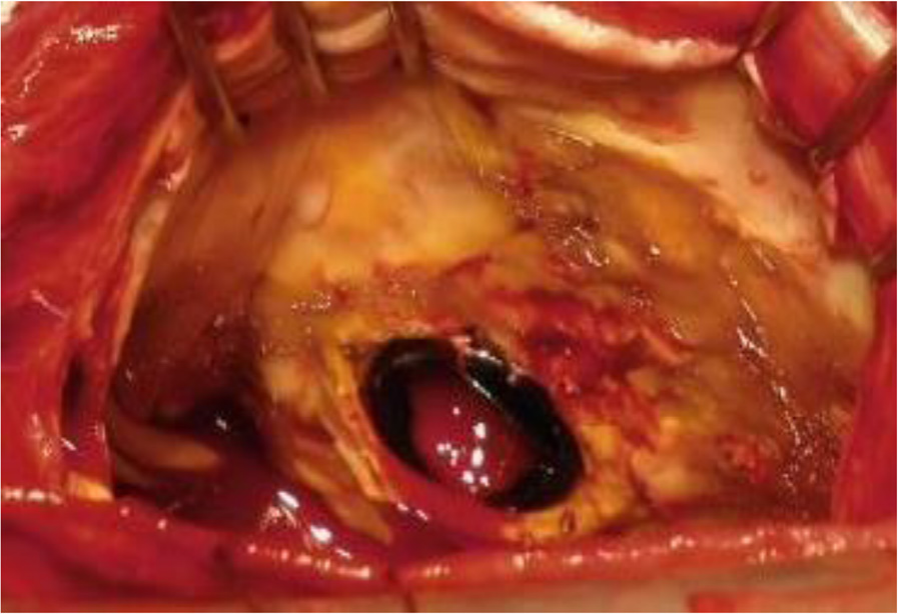
Operative findings of the implanted valve. The suture ring of the implanted prosthesis is covered with pannus, although it is not obliterating the valve orifice

The patient obviously required another operation. Standard cardiopulmonary bypass was established. Under cardiac arrest, right-sided left atriotomy allowed visualization of the implanted valve. Pannus formation was apparent on the prosthetic valve ring (Fig. 3), but it did not obliterate the prosthetic valve orifice on either the atrial or ventricular aspects. After removing the valve, the posterior wall of the left ventricle was seen to be associated with thickened endocardium. A 27 mm Bicarbon bileaflet valve (Sorin Biomedica, Saluggia, Italy) was implanted in an intra-annular position. Plication of the left atrium was undertaken along the incisional line and between the mitral posterior annulus and bilateral pulmonary vein orifices. Weaning from the cardiopulmonary bypass was easy, and her postoperative course was uneventful.

Postoperative echocardiography showed a mean pressure gradient of 4.3 mmHg between the left atrium and ventricle with no mitral valve regurgitation. The left atrium was slightly reduced to 71 mm, and the left ventricular end-diastolic/end-systolic dimension was 50/32 mm with an ejection fraction of 65 %. She was doing well after one and a half years after the operation.

## Discussion

Edmunds and coworkers designed the guidelines for reporting outcomes after prosthetic valve replacement, stating that valve-related complications include thromboembolisms, bleeding complications, prosthetic valve endocarditis, and structural and nonstructural prosthetic valve dysfunctions [1]. Nonstructural valve dysfunction may include paravalvular leak with no apparent endocarditis, cusp entrapment by pannus, and others.

Pannus could prevent a prosthetic valve leaflet from functioning well. This complication is found mainly at long-term follow-up evaluations. We earlier reported four patients who required reoperation because of pannus formation [2, 3]. One of the patients who had pannus at the aortic position underwent reoperation at 97 months. In two others, pannus was at the mitral position and in another it was at both the aortic and mitral positions. These three patients had been diagnosed 20–39 years after their initial operations.

Oh and coworkers reported that the mean interval from the previous operation to the diagnosis of pannus at the aortic position that was causing dysfunction was 16.7 ± 4.3 years [4]. The authors noted that the most common etiology for the previous surgery was valve disease associated with prior rheumatic fever. Ellensen and colleagues also reported a series of 27 cases. They showed that women and younger patients had a higher risk of pannus formation at the aortic position [5]. Doshi and coworkers reported a case of intermittent opening of a mitral tilting disc prosthesis 23 years after implantation [6]. They showed a circumferential pannus on the ventricular aspect on the free margin of the disc.

**Fig. 4:**
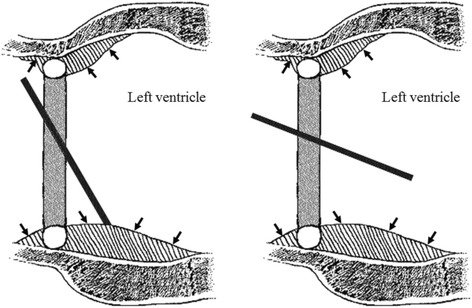
Mechanisms of valve dysfunction of our case. The thickened left ventricular posterior wall associated with pannus growth (*black arrows*) might lead to a stenosis, and it could prevent the disc from closing completely in the case of a tilting disc valve

Pannus overgrowth was recognized in our patient, but it was limited to the suture ring. Preoperative findings indicated prosthetic valve dysfunction with mitral stenosis and regurgitation. Based on the operative findings, we surmised that the posterior wall of the left ventricle had been thickened by a blood flow through the major orifice of the tilting disc. Tilting disc valves do not have laminar, unlike bileaflet prostheses [7]. The thickened left ventricular posterior wall associated with pannus growth might lead to a stenosis, which could prevent the disc from closing completely in the case of a tilting disc valve (Fig. 4). It would be less likely to occur in a bileaflet valve.

In valve disease associated with prior rheumatic fever, the endocardium generally has inflammatory changes, including thickening. Repeated surgeries may also contribute to endocardial thickening.

Pannus that exceeds the suture ring can be resected through the prosthetic valve [8], but subannular pannus or fibrous tissue is not easy to remove. Therefore, valve replacement is an adequate treatment strategy. A bileaflet prosthesis produces parallel laminar flows, and their low profile characteristics can avoid restricted leaflet functioning at the anti-anatomical position of the mitral valve in case of subannular hypertrophic changes.

## Conclusions

Subannular pannus formation and fibrous hypertrophic changes of the posterior portion of the left ventricle can cause mechanical heart valve dysfunction. These changes are particularly observed with a tilting disc valve.

### Consent

Written informed consent was obtained from the patient for publication of this Case report and any accompanying images.

## Additional file

Additional file 1: Left ventriculography. Mitral valve regurgitation from the minor orifice of the Omnicarbon valve is observed. (MOV 4181 kb)
